# The Influence of Empathy Trait and Gender on Empathic Responses. A Study With Dynamic Emotional Stimulus and Eye Movement Recordings

**DOI:** 10.3389/fpsyg.2020.00023

**Published:** 2020-01-31

**Authors:** Eduardo S. Martínez-Velázquez, Alma L. Ahuatzin González, Yaira Chamorro, Henrique Sequeira

**Affiliations:** ^1^Laboratorio de Psicofisiología, Facultad de Psicología, Benemérita Universidad de Puebla, Puebla, Mexico; ^2^Laboratorio de Neuropsicología y Neurolingüística, CUCBA, Mexico Institute of Neuroscience, University of Guadalajara, Guadalajara, Mexico; ^3^University of Lille, CNRS, CHU Lille, UMR 9193 - SCALab - Sciences Cognitives et Sciences Affectives, Lille, France

**Keywords:** empathy, gender, alexithymia, eye movements, dynamic emotional stimulus, arousal

## Abstract

Previous studies have suggested that empathic process involve several components such as cognitive empathy, affective empathy, and prosocial concern. It has also been reported that gender and empathy trait can influence empathic responses such as emotional recognition, which requires an appropriate scanning of faces. However, the degree to which these factors influence the empathic responses, which include emotion recognition, affective empathy, and cognitive empathy, has not yet been specified.

**Aim:** The aim of the present study was to identify the differences between individuals with high and low level of empathy trait, as well as differences between men and women, in an explicit task in order to evaluate the empathic responses.

**Methods:** With this goal in mind, we recorded eye movements during the presentation of dynamic emotional stimuli (joy, anger, fear, and neutral videos). After watching each video, participants had to rate the valence and arousal dimensions of emotional content and explicit empathy responses were assessed. Thirty participants (15 women) were included in a High Empathy group (HE; mean age = 21.0) and 30 participants (16 women) in the Low Empathy group (LE; mean age = 21.2), according to their scores in the Interpersonal Reactivity Index (IRI) scale.

**Results:** As expected, the HE group showed higher scores than the LE group in the explicit empathy responses. These differences, based on global scores, were mainly explained by affective empathy and cognitive empathy responses but not by emotional recognition one. No differences were observed by gender in these measures. Regarding eye movements in the dynamic emotional stimuli, HE group had longer fixation duration on the eyes area than LE group. In addition, women spent more time on the eyes area in comparison to men.

**Discussion:** Our findings suggest that both men and women with high empathy trait are more accurate to empathizing but not on the basis of the emotional recognition response. The fact that women spent more time on the eyes area did not seem to affect the empathic responses to the dynamic emotional stimulus. Overall, empathic responses of both men and women are modulated by their empathic trait. In addition, empathic trait and gender seem to impact strategies to deal with emotional facial information.

## Introduction

Empathy is the ability to understand and to share the internal states of others (Christov-Moore et al., 2014; Noten et al., 2019). Although an agreement on the concept of empathy is not clearly found in the literature, most researchers agree that it involves a multidimensional process that includes three basic elements: affect sharing, mentalizing, and prosocial concern (Christov-Moore et al., 2014; Noten et al., 2019). *Affect sharing* means vicariously sharing targets’ internal states between one and others. Some authors also named it emotional contagion and relate it with the tendency to automatically mimic and synchronize facial expressions, vocalizations, postures, and movements with those of another person. *Mentalizing* is the propensity to adopt the perspective of others, it involves the ability to explicitly reason and draw inferences about their mental states (Zaki and Oschner, 2012). Mentalizing has been associated with other concepts like mind theory and perspective taking (PT) (Davis, 1980; Singer, 2006; Walter, 2012). The last component is *prosocial concern*, which underlies the emotional regulation and involves the ability to distinguish one’s own emotions from others. It is related with motivation that people have to act when helping others (Decety and Jackson, 2006; Singer, 2006; Zaki and Oschner, 2012).

Other authors have classified the previous components in affective empathy (*affect sharing*), cognitive empathy (*mentalizing* or perspective taking), and prosocial behavior skills (Retuerto, 2004; Calvo et al., 2008; Zaki and Oschner, 2012; Balconi and Canavesio, 2014). In addition, another aspect related with the empathic process concerns the facial emotional recognition; this gives the possibility to decode others’ internal states from facial expressions (Hall and Masumoto, 2004; Balconi and Canavesio, 2014). Thus, the empathic responses present the interest to allow us to react in the most socially appropriate way in order to interact successfully with others in the daily life (Singer, 2006) including social networks (Schmaelzle et al., 2017). In summary, the empathic process is important for social interactions and involves affective empathy (*sharing*), cognitive empathy (*mentalizing*) linked with facial emotional recognition, and behavioral aspects like prosocial concern.

Regarding the process of facial emotional recognition, it has been suggested that it is related with a specific visual scan pattern, in which those who look at the eyes for a longer period of time show greater accuracy and speed to recognize emotions than those who spent less time looking on eyes’ area (Hall and Masumoto, 2004; Calvo et al., 2008; Balconi and Canavesio, 2014). Some studies have reported participants spent more time looking at the eyes area in emotionally stimulus than neutral, highlighting the notion that eye-to-eye encounters are critical to successful engage social interactions (Mason et al., 2005; Cowan et al., 2014). In addition, some clinical abnormalities in socially directed eye-gaze patterns to facial features exhibit low-emotional empathy, such as schizophrenia, or autism (Klin et al., 2002; Joshua and Rossell, 2009). In this context, it has been reported that empathy traits and gender can influence the visual scanning linked to emotional recognition (Balconi and Canavesio, 2014; Cowan et al., 2014; van Rijn et al., 2014). Otherwise, empathy traits have been described as the qualities or the empathic tendencies that people identify in themselves (Hoffman, 1977; Davis, 1980). They are usually determined by using self-reported scales such as the Interpersonal Reactivity Index (IRI) (Davis, 1980) or the *Balanced emotional empathy scale* (BEES), among others (Hemmerdinger et al., 2007). Thus, some authors have related empathy traits with the emotional recognition as evaluated through facial scanning (Balconi and Canavesio, 2014).

An important study reported the influence of social empathy on processing of emotional facial expressions (Balconi and Canavesio, 2014). Using the BEES scale, participants were classified in two groups, with high and low empathy traits. Then, positive and negative facial statics stimuli were presented to participants who had to categorize each emotional expression (Balconi and Canavesio, 2014). The high-empathy group showed shorter reaction times, longer durations, and greater number of fixations on the eyes and mouth regions than those obtained in the low-empathy group. These differences were mainly observed in faces showing emotions of joy, fear, and anger (Balconi and Canavesio, 2014). The authors concluded that empathy trait, when assessed through eye movements, may have a significant impact on cognitive and attentional processes of emotional facial expressions. However, in this study, the influence of gender was not determined.

In this regard, it has been reported that women are faster and more accurate to recognize emotional expressions than men, especially emotional expressions of joy, anger, and surprise (Hall and Masumoto, 2004; Hall et al., 2010). This advantage has been related to the fact that women show longer fixations on the eyes’ region of faces than men (Hall et al., 2010). However, only one of these studies determined the participants’ empathy trait, and no gender differences were reported (Hall et al., 2010). In the remaining studies, where empathy trait was not assessed, it is not clear at what point it influences visual scanning and emotional recognition. This is particularly relevant when considering studies reporting that empathy trait is usually higher in women than men (Davis, 1980; Riggio et al., 1989; Mestré-Escrivá et al., 2004). In addition to the fact that studies did not consider together empathy trait and gender, the use of static stimulus constitutes another limitation for several studies about empathic processes. Indeed, it has been suggested that the use of repetitive static stimulus is a potential source of fatigue and habituation that might have limited the ecological value of their findings (Balconi and Canavesio, 2014). On the contrary, dynamic stimuli exalt natural emotional expressions, which allows to evoke empathic responses (including emotional recognition), and to evaluate empathic processes more accurately (Regenbogen et al., 2012; Cowan et al., 2014).

In this line, we have not found studies that examine the influence of both, empathy trait and gender, on the empathic process using dynamic stimuli in typical population. Although some studies have evaluated the empathic process using dynamic stimulus and recorded eye movements in clinical populations (van Rijn et al., 2014; van Goozen et al., 2016; Hubble et al., 2017; van Zonneveld et al., 2017), they did not determine the influence of empathy trait or gender. As far as we know, only one study has included typical adult population, empathy trait, and dynamic stimuli (video) to compare neutral and sad emotions (Cowan et al., 2014). The researchers reported a positive correlation between the subject’s levels of empathy concern (evaluated by IRI scale) and the fixation duration on the eyes’ region of the emotional stimulus. Besides, they reported that subjects with high levels of empathy concern were more accurate in recognizing emotion facial expressions. They thus suggested that the empathic level predicts the ability to recognize emotional facial expression. It is important to note that participants in this study were all women; hence, the trait of empathy in men is not known nor the gender differences. Therefore, the role of gender on the empathic process is not yet clear.

The aim of present study was to compare empathic responses (emotional recognition, affective, and cognitive empathy linked with prosocial behavior), using dynamic emotional stimuli (joy, anger, fear, and neutral) and eye movement recordings, in men and women having low and high levels of empathy trait. Empathic trait and responses were evaluated by self-report scales and an empathy test, respectively. Our main hypothesis was that participants with high empathy trait will exhibit higher scores on empathic responses and longer fixations on the eyes than participants with low empathy trait, regardless of gender. Moreover, we expected that videos with emotional content would collect longer fixation on the eyes’ region than videos with neutral content, regardless of trait of empathy.

## Materials and Methods

This study was approved by the Ethics Committee of the Faculty of Psychology of the Autonomous University of Puebla (BUAP), in agreement with the ethical norms that regulate the professional, scientific, and academic practice of Psychology in Mexico (Sociedad Mexicana de Psicología, 2017). All participants gave written informed consent in accordance with the Declaration of Helsinki.

### Participants

Sixty undergraduate students (29 men) of the BUAP participated in the study. Participant’s age ranged from 18 to 30 years (men’s age *M* = 21.1, SD = 2.4; women’s age *M* = 20.9, SD = 1.8). Participants were pre-selected from a group of students (*n* = 714) who answered the Interpersonal Reactivity Index (IRI-adaptation) (Ahuatzin et al., 2019). They were assigned to two different groups according to their scores in the IRI scale: (1) High empathy trait (HE, *n* = 30) and (2) Low empathy trait (LE, *n* = 30). Significant differences for global scores of the empathy scale (IRI) were observed between HE and LE groups [*t*(58) = 21, *p* = 0.001, *d* = 5.42]. The analysis of IRI’s sub-scales revealed differences between both groups in the Empathic Concern (EC), a sub-scale of the affective component [*t*(58) = 15.40, *p ≤* 0.001, *d* = 3.97] and in the Perspective Taking (PT), a sub-scale of the cognitive component [*t*(58) = 25.78, *p* = 0.001, *d* = 6.65], as established in the inclusion criteria. Empathy groups did not differ by age [*t*(58) = −0.43, *p* = 0.66] or gender distribution (*χ*^2^ = 0.67, *p* = 0.79). Visual impairment, history of neurological events, substance abuse, and psychotic symptoms requiring pharmacological treatment were retained as criteria of exclusion.

### Empathy and Alexithymia Scales

#### Interpersonal Reactivity Index Scale

Trait empathy was assessed by an adaptation of Interpersonal Reactivity Index, conducted with a sample of university students (Ahuatzin et al., 2019), which has shown good reliability indices (*α* = 0.81). We used the IRI scale because it evaluates individual differences in empathic trends from a multidimensional point of view. The instrument is composed of 28 items grouped into four sub-scales, which measure the affective (empathic concern, EC; personal distress, PD) and the cognitive (perspective taking, PT; fantasy, F) components. As previously reported, responses to the EC sub-scale are considered as a measure of the emotional empathy trait and those of the PT sub-scale as a measure of the cognitive empathy trait (Cowan et al., 2014). The IRI score cut-offs for each group was established as follows: one standard deviation above the mean for the high empathy trait group (HE) and one standard deviation below the mean for the low-empathy trait group (LE) according to the adapted version (Ahuatzin et al., 2019). For both groups, range values were different for men and women: to HE group, women (36–40 points to EC and 31–35 points to PT component) and men (33–40 points to EC and 29–35 points to PT component); to LE group, women (10–28 points to EC and 7–19 points to PT component) and men (8–23 points to EC and 11–17 points to PT component).

#### Toronto Alexithymia Scale

Considering that previous research has linked alexithymia trait with empathy trait (Moral and Ramos-Basurto, 2015; Martínez-Velázquez et al., 2017), the alexithymia trait was assessed by using Spanish version (Moral, 2008) of the Toronto Alexithymia Scale (TAS-20). This version has shown good reliability (*α* = 0.82) and stability indices in the Latin American population. The TAS-20 is composed of 20 items divided into three categories: (1) difficulty expressing feelings (5 items); (2) difficulty in identifying feelings (7 items) and, (3) externally oriented thinking that contains 8 items.

### Stimuli

Based on the methodology proposed by Cowan et al. (2014), we created our own dynamic stimuli, with an actress (of approximately the same age as the target sample) who was unknown to the participants. The actress was instructed to look directly to the camera as she spoke and being as spontaneous as during a daily-life conversation. We created four videos of 180 s each. Each video began with the presentation of a fixation point (a white cross of 2 cm) in the center of the screen (black background) for 2 s, then the actress appeared and started to tell fictitious personal event with a specific emotional valence: joy, anger, fear, or neutral. In the emotional videos, the actress was requested to be more expressive than in the neutral one.

All videos were presented on a 14-inch monitor with a resolution of 1,280 × 720 pixels. The image of the video was presented in the center of the screen and had a size of 11.5 cm in height and 20.5 cm in length. These videos were previously validated in a pilot study with open population (168 participants). Each video was identified as being strongly related (%) to an emotional or a neutral content: joy (90%), angry (90%), fear (80%), and neutral (80%). Emotion arousal of each video was rated with the SAM scale (Self-Assessment Manikin; Bradley and Lang, 1994) ranging from 1 (very calm) to 9 (very aroused). In the pilot study, mean arousal scores were respectively 9.2 for joy, 9.1 for angry, 9.6 for fear, and 6.2 for neutral (Ahuatzin, 2018).

### Empathic Responses, Emotional Measures, and Eye-Movement Recordings

To evaluate empathic responses, corresponding here to emotional recognition, we used an explicit empathy test, according to the standards of previous studies presenting dynamic stimuli (van Goozen et al., 2016; Hubble et al., 2017). Participants were asked to identify the emotional context in each video and their responses were scored on the basis of three components: (1) Recognizing the most important emotion in the actress: this component has been considered as cognitive empathic response (van Goozen et al., 2016). If the participant identified the main emotion more intensely than other emotions on the list, they received three points. If they indicated it as the second strongest, they got two points. If they pointed out two emotions with the same intensity, they received only one point. If they did not identify the emotion at all, they obtained zero points. (2) Feeling emotions concurrent or similar to the speaker expressed on the video (affective empathy). If the participant wrote a congruent or similar emotion, two points were awarded. If the participant generated a different emotion he/she get a point and zero points when he/she did not report any emotion. (3) Additionally, to understanding the situation, the participants have to say the reasons (cognitive empathy) for which the actress felt that way. This question was rated with three points if the reason they gave included a direct reference to the emotions of the actress, two points if in the answer was referred to the situation and not to emotions of actress. A point was assigned if the answer makes reference only to participant emotion’s without taking into account the experience of the actress. Finally, it was scored 0 if the participant gave an irrelevant answer, e.g., “I thought it was boring.” The maximum score was 8 by each video. In addition, and in order to evaluate the emotional value of each video, we used the classical nine-point SAM scale (Self-Assessment Manikin; Bradley and Lang, 1994), based on valence and arousal dimensions of emotion. The valence value was scored from 1 (very unpleasant) to 9 (very pleasant) and the arousal was scored from −4 (very calm), to 4 (very aroused).

Binocular eye movements were recorded during video presentation using an infrared based video tracking (Tobii Pro X2-30 Technology). This eye tracker operates with a sampling rate of 30 Hz, and a spatial error < 0.01. Tobii Studio (3.0) software was used to present the videos, record eye movements, and conduct the off-line analyses to extract the measure of fixation duration within specific Areas of Interest (AOIs).

Considering previous studies of emotion recognition in faces, we established two main AOIs: eyes and mouth (Cowan et al., 2014). To determine the dynamic AOIs, we drew rectangular and elliptical areas using Tobii Studio software on each video, having the same size, for both emotional and the neutral conditions. We attempted to be as precise as possible with regard to the objects of interest (delimited by using the upper/lower and the left/right boundaries) and, particularly, defining blank spaces between AOIs as proposed by Holmqvist et al. (2011). We used the default Tobii Studio threshold for minimal fixation duration (80 ms).

### Procedure

**Figure 1:**
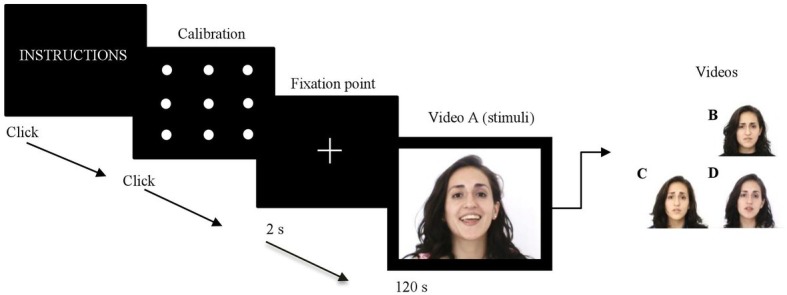
Sequential steps of one trial. Duration of the fixation cross (2 s) and the presentation of each video (120 s). Content of video stimuli: A (joy), B (anger), C (fear) and (D) neutral. The presentation of videos was counterbalanced.

The candidates whose scores achieved the established thresholds for HE and LE groups were invited to participate in the study: one experimental session of approximately 50 min, carried out in a quiet room of the university. The session includes three successive parts. Firstly, a brief interview in order to corroborate the remaining inclusion criteria, the participants answered to the TAS-20 and a visual test was conducted to ensure the participants will not have visual problems. Secondly, instructions were given before starting the experiment on the screen and participants were told that they have to look the videos and then we would ask them some questions about it. No further background information was provided about the videos in order to allow a naturalistic viewing. Participants were seated in front of eye-tracking system at a distance of 60 cm, wearing headphones. Before the video presentation, a nine-point calibration was conducted. For each trial, participants first had to fixate their eyes on a central cross, then the video (see Figure 1). Finally, at the end of each video, the participant was debriefed about the video content through the explicit empathy test and the SAM to rate the emotional value of the visualized video. The presentation of emotional and neutral videos was counterbalanced across participants.

### Statistical Analysis

Independent *t*-tests were conducted to analyze differences between groups in age, and in scores for the IRI and TAS scales, and the arousal and valence dimensions. Chi-square was used to estimate differences of gender distribution. We also performed correlation analyses (Pearson’s coefficient) between IRI and TAS scores.

To analyze the effect of empathic responses, gender, and type of emotion over the score of explicit empathy test, we conducted an ANOVA with three independent factors: Group × Gender × Emotion. Partial eta squared (*η*^2^) was used as an index of effect size.

To determine the effect of empathy trait, gender, and type of emotion on the eye movement measure (fixation duration), a mixed-factorial analysis of variance (ANOVA) was carried out: Group (HE and LE) and Gender (men and women) were introduced as inter-subject factors; emotions (joy, anger, fear, and neutral) and AOIs (eyes and mouth) were considered as intra-subjects factors. Greenhouse-Geisser correction was used and Bonferroni *post hoc* test was applied in all analyses. Eta-squared (*η*^2^) test was used to estimate the effect size. Statistical analyses were conducted using SPSS v22. Power analysis was performed with G*Power (Faul et al., 2007). *Post hoc* sensitivity power analysis showed that the sample of this study had sufficient power (*β* = 0.80) at a significance level of *α* = 0.05 to detect medium to large effect sizes for an ANOVA with fixed effects (*F* ≥ 1.54) and to detect medium effect sizes in a MANOVA with between-within interactions (*F* ≥ 2.77).

## Results

### Clinical Characteristics of Participants

LE group presented significant higher TAS-20 scores than the HE one [*t*(58) = −4.94, *p* ≤ 0.001, *d* = −1.27]. Additionally, for all participants, a negative correlation was observed between global scores of the IRI and the TAS [*r*(58) = −0.58, *p* ≤ 0.001]. This correlation remained significant when analyzing separately men [*r*(58) = −0.56, *p* ≤ 0.001] and women [*r*(58) = −0.47, *p* ≤ 0.001].

### Emotional Values of Videos

Concerning the valence evaluation of videos, higher scores were observed in the HE group than the LE group for videos of joy [*t*(58) = 2.11, *p* < 0.05] and fear [*t*(58) = 2.27, *p* < 0.05]. When analyzing valence by gender, no differences were observed. Mean scores of the arousal evaluation of videos were higher in HE than in LE group (*p* < 0.05) in the joy condition [*t*(58) = 2.97, *p* < 0.05] but not in the other emotional or neutral conditions. Regarding the gender, the analysis showed that only women presented significant differences [*t*(58) = 3.58, *p* < 0.05], with a higher score in the HE than in LE group for joy and fear videos. No differences were observed in any other condition. Mean arousal and valence values for empathy groups, for different videos, and for women and men are summarized in Table 1.

**Table 1:** Descriptive values of valence and arousal for emotional and neutral videos by high and low empathy trait (HE and LE) and by gender.

		Valence						Arousal					
		Total		Women		Men		Total		Women		Men	
		*M*	SD	*M*	SD	*M*	SD	*M*	SD	*M*	SD	*M*	SD
HE	Joy	5.0^*^	2.4	4.8	2.7	5.2	2.1	2.8^*^	1.1	3.1^*^	0.9	2.5	1.4
	Angry	4.3	2.2	4.3	2.6	4.2	1.8	−1.8	1.7	−2.0	1.6	−1.5	1.7
	Fear	5.1^*^	2.6	5.1	2.9	5.0	2.2	−2.23	1.6	−2.5^*^	1.5	−1.8	1.7
	Neutral	2.4	1.8	2.3	1.9	2.4	1.8	−0.8	1.7	−0.7	2.0	−1.0	1.3
LE	Joy	3.8^*^	2.2	3.7	2.4	3.8	2.0	1.9^*^	1.3	1.6^*^	1.5	2.2	1.0
	Angry	3.5	1.6	3.6	1.6	3.3	1.6	−1.4	1.3	−1.3	1.4	−1.5	1.1
	Fear	3.7^*^	1.9	3.6	1.8	3.9	2.1	−2.23	1.3	−1.8^*^	1.5	−2.4	1.1
	Neutral	2.6	1.9	3.1	2.5	2.2	1.2	−1.0	1.6	−1.3	1.7	−0.7	1.5

*HE, high empathy; LE, low empathy*.

*Mean and SD of score of Self-Assessment Manikin by group and gender to each condition*.

* *p < 0.05*.

### Empathic Responses to Videos

The ANOVA showed significant main effects of empathy groups over the total score of the explicit empathy test [*F*(1,56) = 45.83, *η*^2^ = 0.45, *p* ≤ 0.001]. *Post hoc* analysis showed that the HE group showed higher scores than the LE group (*p* < 0.05). No main effects of gender [*F*(1,56) = 0.97, *η*^2^ = 0.01, *p* = 0.32] or the type of emotion [*F*(3,56) = 0.61, *η*^2^ = 0.01, *p* = 0.60] were observed. There were no significant interactions between factors group × gender [*F*(1,56) = 2.38, *η*^2^ = 0.04, *p* = 0.12], group × emotions [*F*(3,56) = 1.15, *η*^2^ = 0.02, *p* = 0.33], or emotions × group × gender [*F*(3,56) = 1.70, *η*^2^ = 0.02, *p* = 0.17].

Regarding the analysis of empathy responses, by components, we did not find main effects of group in the Emotional recognition component [*F*(1,56) = 0.45, *η*^2^ = 0.008, *p* = 0.50], gender [*F*(1,56) = 0.03, *η*^2^ = 0.001, *p* = 0.84], or emotion [*F*(3,56) = 0.76, *η*^2^ = 0.013, *p* = 0.49], or any interaction, including group × gender [*F*(1,56) = 0.038, *η*^2^ = 0.001, *p* = 0.84]. However, in the Empathic correspondence component, which is related to the affective empathy, a main effect of group was observed [*F*(1,56) = 24.57, *η*^2^ = 0.30, *p* ≤ 0.001] but not of gender [*F*(1,56) = 3.32, *η*^2^ = 0.05, *p* = 0.07], the type of emotion [*F*(3,56) = 0.34, *η*^2^ = 0.006, *p* = 0.77] or interaction group × gender [*F*(1,56) = 1.28, *η*^2^ = 0.02, *p* = 0.26] nor any other. Likewise, in the Empathic reason scores we found a main effect of group [*F*(1,56) = 75.30, *η*^2^ = 0.57, *p* ≤ 0.001] and a significant interaction group × gender × emotions [*F*(3,56) = 3.33, *η*^2^ = 0.05, *p* < 0.05]. The *post hoc* analyses revealed higher scores in the HE compared with LE group of men in each condition (joy, angry, fear, and neutral) (*p* < 0.05). Similar results were observed in the HE group of women compared with LE group of women in joy, fear, and neutral conditions (*p* < 0.05), but not in angry one (*p* = 0.35). In concordance with these results, we found that LE group of women presented higher scores than LE group of men in angry condition. Additionally, only the LE group of women presented higher scores in joy than angry (see Table 2). Mean scores of empathic test to different videos by empathic group and gender are summarized in Table 2.

**Table 2:** Descriptive scores of empathic components for emotional and neutral videos by high and low empathy trait (HE and LE) and by gender.

		Emotional recognition score						Empathic correspondence score						Empathic reason score					
		Total		Women		Men		Total		Women		Men		Total		Women		Men	
		*M*	SD	*M*	SD	*M*	SD	*M*	SD	*M*	SD	*M*	SD	*M*	SD	*M*	SD	*M*	SD
HE	Joy	2.5	0.7	2.5	0.8	2.5	0.7	1.6^*^	0.5	1.6	0.4	1.5	0.6	2.1^*^	0.9	2.3^*^	0.5	1.8^*^	1.1
	Angry	2.3	0.9	2.1	1.0	2.5	0.7	1.4^*^	0.7	1.5	0.6	1.4	0.8	1.8^*^	1.0	1.5	0.9	2.0^*^	1.0
	Fear	2.2	0.8	2.4	0.8	2.0	0.9	1.5^*^	0.5	1.4	0.6	1.5	0.5	1.9^*^	0.8	1.6^*^	0.7	2.1^*^	0.9
	Neutral	2.3	1.1	2.2	1.1	2.4	1.1	1.7^*^	0.5	1.8	0.4	1.5	0.6	2.0^*^	0.7	1.8^*^	0.6	2.1^*^	0.8
LE	Joy	2.4	0.9	2.2	1.0	2.6	0.8	0.9^*^	0.7	1.2	0.6	0.6	0.8	0.6^*^	0.9	0.6^*^	1.0	0.5^*^	0.9
	Angry	2.2	0.9	2.4	0.8	2.0	1.0	1.2^*^	0.8	1.3	0.7	1.0	0.9	1.2^*^	0.8	1.2	0.7	0.5^*^	0.7
	Fear	2.0	0.9	2.1	0.8	1.8	1.0	1.0^*^	0.7	1.3	0.6	0.8	0.8	1.0^*^	0.8	1.0^*^	0.8	0.6^*^	0.8
	Neutral	2.4	0.8	2.2	0.9	2.6	0.8	1.0^*^	0.7	1.1	0.5	0.9	0.9	0.6^*^	0.7	0.6^*^	0.7	0.4^*^	0.7

*HE, high empathy; LE, low empathy*.

*Mean and SD of score of empathy test by group and gender to each component*.

* *p < 0.05*.

### Eye Movements to Videos

The results of mixed ANOVA showed a tendency to report longer fixation durations in the HE group than the LE group on both AOIs (eyes and mouth) [*F*(1,56) = 3.189, *η*^2^ = 0.54, *p* = 0.07]. No differences were found in gender [*F*(1,56) = 4.82, *η*^2^ = 0.079, *p* < 0.05]. A main effect of emotional valence was observed [*F*(3,56) = 6.66, *η*^2^ = 0.106, *p* < 0.05]: *post hoc* comparison revealed that fixations were longer in the video with joy content in comparison to angry (*p* < 0.05) and neutral condition (*p* < 0.05). The duration of fixations was shorter in the angry condition in comparison to fear and joy videos (*p* < 0.05). Differences by AOIs were also observed [*F*(1,56) = 317.94, *η*^2^ = 0.85, *p* < 0.05]: longer fixation durations for eyes’ AOI when compared to the mouth’s AOI (*p* < 0.05) in all participants.

**Figure 2:**
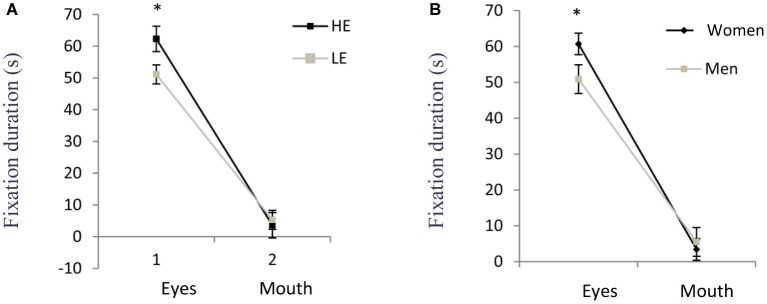
Total duration of fixations (s) on facial areas of interest (Eyes, Mouth) by group (**A**: HE and LE) and by gender (**B**: Women and Men). *p < 0.05.

Regarding interactions among factors, empathy groups and AOIs showed a significant interaction [*F*(1,56) = 4.59, *η*^2^ = 0.076, *p* < 0.05]: the HE group exhibited longer fixation duration than the LE group but only on the AOI of eyes (*p* < 0.05). There also was an shared effect of AOI × gender [*F*(1,56) = 4.82, *η*^2^ = 0.079, *p* < 0.05]: women presented longer fixation durations than men on the eyes’ AOI (*p* < 0.05) (see Figure 2). Finally, an interaction AOI × emotions was observed [*F*(3,56) = 5.77, *η*^2^ = 0.094, *p* < 0.05]: longer fixation durations were reported on the eyes area when analyzing the videos with joy content, in comparison to videos with angry and neutral contents (*p* < 0.05). There were no more significant interactions.

## Discussion

The aim of this study was to identify the influence of empathy trait and gender on empathic responses. To this end, these responses and eye movement measures have been recorded to the presentation of emotional and neutral dynamic stimuli in the form of videos. Based on IRI’s scores, two groups of high and low level of empathy trait, respectively, HE and LE groups, were constituted. Each group integrates a similar number of women and men without differences in terms of age and education level. In addition, on the basis of previous identified links between alexithymia and empathy traits, alexithymia was assessed, thanks to the TAS-20 scale.

This experiment brings interesting new data supporting hypotheses related to empathy trait and gender. Firstly, empathic responses were higher in HE group than in LE group; furthermore, HE group had longer fixation duration on the eyes’ area than the LE group; in addition, high scores in alexithymia were observed in LE group. Secondly, women spent more time looking to the eyes’ area in comparison to men. Finally, neither the empathy trait, nor gender modulates the recognition of emotional dynamic stimuli.

### Empathy Trait Effects

#### Empathic Responses

Present findings suggest that the empathy trait, evaluated by self-report, affects the empathic processes related to affective and cognitive empathy when using emotional dynamic stimuli. These results differ from those reported by van Zonneveld et al. (2017) showing a diminished affective and a normal cognitive empathy in children with high risk of developing criminal behavior. However, in addition to a potential population effect, the authors consider the affective empathy by applying physiological measures and did not analyze cognitive and affective components on the basis of the empathy test, contrary to suggestions made by several authors (van Rijn et al., 2014; van Goozen et al., 2016; Hubble et al., 2017).

Contrary to affective and cognitive empathic responses, no effect was observed in the emotional recognition of stimuli. In this context, previous studies have reported differences between high- and low-empathy trait, taking into account the response speed and the discrimination between emotions in static stimuli (Braaten and Rosén, 2000; Hall and Masumoto, 2004; Balconi and Canavesio, 2014). In our experiment, additional elements integrating dynamic stimuli, such as facial expression, tone of voice, and the content of the story, could explain observed differences in comparison to static stimuli. However, contradictory results have been reported from studies using emotional dynamic stimuli; particularly, an unimpaired emotional recognition had been reported in clinical populations and controls using dynamic stimuli (van Goozen et al., 2016; van Zonneveld et al., 2017). In brief, such contradictory results could be more related to the difficulty to consider the concept of emotional recognition than to the composition of emotional stimuli. Indeed, a study that assessed adolescents with diagnosis of conduct disorder (van Goozen et al., 2016), reported low scores in affective empathy to a video with fear content, when compared to those obtained in respective control groups. The authors interpreted these differences on the basis of the difficulties to empathize, but not in terms of emotional recognition.

As far as we know, another study used dynamic stimuli and reported impairments in empathy responses, including the emotional recognition component, in a clinical population. In effect, the authors compared the empathic responses between patients Klinefelter Syndrome’s patients and a control group, using dynamic emotional expressions (van Rijn et al., 2014). The authors reported that the Klinefelter Syndrome subjects showed diminished empathic responses including the three components: affective empathy, cognitive empathy and emotional recognition. However, it is to note that the main symptom associated with Klinefelter syndrome is a cognitive dysfunction (Lee et al., 2010; van Rijn et al., 2014), unlike the two previous studies where the clinical populations manifested social difficulties as the main symptom (van Goozen et al., 2016; van Zonneveld et al., 2017). Overall, further studies are clearly needed to disentangle empathic responses to emotional dynamic stimuli. Perhaps, the association of empathy test with physiological measures, especially those related with emotional activation (Martínez-Velázquez et al., 2017), could originate more heuristic results.

#### Eye Movement Measures

The time spent exploring the videos was differently distributed in groups of empathy level: the HE group spent more time looking at the eyes area than the LE group, whereas no differences between groups were observed in the mouth area. We also observed that the HE group had longer fixations in emotional and neutral conditions in comparison to LE group. Our experiment confirms the relation between empathy trait and fixation durations previously reported in a sample including only women (Cowan et al., 2014). Furthermore, as these authors, we observed longer durations of fixation in joy condition than in angry one. This is quite coherent with the dimensional theory (Lang et al., 1993) proposal, which postulates an approach behavior facing pleasant information and an avoiding reaction to unpleasant stimulation.

In this context, it has been suggested that through the eyes, a greater synchronization of affective states takes place in face-to-face encounters, thus promoting the quality of our social interactions (Cowan et al., 2014). These findings add evidence, *via* eye movements, of the differences between groups with different empathy traits when analyzing dynamic stimulus with emotional content.

#### Alexithymia Level

As indicated, we also observed that participants of LE group exhibited a high score in alexithymia, a difficulty to identify, analyze, and express emotional experiences (Sifneos, 1973; van der Velde et al., 2013) widely related to deficits in social skills (Goerlich et al., 2012; Martínez-Velázquez et al., 2017). In fact, the observed negative association between empathy trait and alexithymia levels fits well with observations reported in previous studies (Fossati et al., 2009; Moral and Ramos-Basurto, 2015). Our findings support the contrasting relation between both variables, in men as in women and suggest that results of LE group could be influenced by alexithymia scores. Particularly, in arousal judgments, we found that HE group perceived emotions of joy and fear with higher intensity than LE group. In addition, we observed that the HE group, in comparison to the LE group, identified with higher pleasure the content of joy and with higher displeasure the content of fear in comparison to the LE group, as observed in their SAM scores (Bradley and Lang, 1994). These findings suggest that LE group has a higher threshold to generate emotional arousal in both emotions (joy and fear) and lower level for differentiating pleasant-unpleasant conditions than HE group. This trend is in line with alexithymia symptoms (Sifneos, 1973; Bagby et al., 1994). Present results support previous studies showing the need to understand better interactions between empathy trait and alexithymia level in order to recognize emotional information.

Considering the above, we speculate that difficulties in social interaction linked with low empathy trait may be related to impairments of affective and cognitive empathy but not to emotion recognition. In other words, individuals expressing a LE trait could identify emotions but seem unable to share and adopt the affective perspective of others. This could explain in part their difficulty to assume prosocial behaviors and, by this way, potentially increase clinical risks as it has been reported in previous studies (van Zonneveld et al., 2017).

### Gender Effects

#### Empathic Responses

Our findings showed an influence of gender only on cognitive empathy component in the angry condition. In this sense, the men were clearly different between low- and high-empathy trait in all emotions, while women had similar responses to anger condition regardless of empathy trait. A possible explanation may be due to the fact that women have higher skills than men to recognize angry (Sokolov et al., 2011) and, apparently, it is independent of the level of empathy. In this regard, the same study reported that females are more accurate than males in recognizing angry bodies (Sokolov et al., 2011), which is consistent with an evolutionary and cultural point of view, linked with threat-avoidance goals (Krüger et al., 2013). Moreover, some studies have reported that women have an advantage in emotional recognition (Hall and Masumoto, 2004). Nevertheless, as described above, they used a different methodology (static stimuli). As far as we know, our study is the first one to use dynamic stimuli to explore the differences by gender and empathy trait with open population.

#### Eye Movement Measures

Concerning the influence of gender on eye movements, both men and women spent more time fixating the eyes than the mouth, but this difference was more pronounced in women. This fact, consistent with previous studies (Hall and Masumoto, 2004), suggests that women and men have a different mechanism to process dynamic emotional stimuli. In addition, we found that the women spent more time on joy videos compared with neutral, while the men spent less time on anger in comparison than joy and fear. According to evolutionary and cultural point of view, the present findings are consistent with evidence that in case of men, it seems that they tried to avoid looking at the angry condition in order to diminish the probability to engage a situational conflict (Krüger et al., 2013).

Although the time spent looking at the eyes is important for the processes of empathy, as was observed in the results of the HE group in the present study, the fact that women fixated for longer time on the eyes than men does not guarantee an adequate empathic response, as was evidenced by the LE group of women. Some authors have suggest that some regions of the face provide early important information about the emotional meaning of a stimulus, such as the eyes, as they seem to be fixated in the first instance, while other emotional characteristics become relevant at later stages of the visual inspection (Balconi and Canavesio, 2014; van Rijn et al., 2014). Thus, when dynamic stimuli are used, the participants have access to non-facial cues, such as tone of voice, gestures, and contextual information. This may have rendered the information from the face less important (Hubble et al., 2017). The time spent in the eyes may be relevant to distinguish between a fast capture of emotional information and a late, accurate perception of same information (Hall and Masumoto, 2004; Balconi and Canavesio, 2014). Thus, in the case of dynamic stimulus, the time employed to analyze the eyes may help also to conjugate early and late processes to recognize emotions (Cowan et al., 2014), but this advantage, observed especially in women, does not seem to depend on high or low level of empathy trait. Furthermore, the fact that there were no correlations between empathic responses and duration of fixations, neither in the present study, nor in a previous one (Hubble et al., 2017), supports the idea of a capture process of facial emotions little connected with empathic responses.

## Limitations and Conclusions

In order to overcome the limitations of static emotional stimuli, we combined for the first time the presentation of dynamic emotional stimuli and the recording of eye movements in a research aiming to explore effects of empathy trait and gender on empathic responses. In spite of our original approach, sustained by a rigorous methodology using standardized videos instead of classical static photographs, we have to recognize some limitations. First, the difficulty to define and operationalize components of empathy could contribute to weaken the interpretation of some results. In the future studies, this limitation could be attenuated by concomitant recordings of empathic responses and physiological indices, in particular those related to the arousal dimension of emotion. In this frame, it could also be interesting to assess the prosocial concern component of empathy. Second, although emotions were clearly identified in each video and results showed high levels of correct emotion identification (joy 90%, angry 90%, fear 80%, and neutral 80%) and high arousal levels, further studies need to integrate new techniques helping to record behavioral and physiological indices linked to more naturalistic stimulations. Indeed, videos remain more artificial when compared to a real-life conversation or social interactions. Nevertheless, recording apparatus, like the eye-tracking techniques or wearing special glasses, quite useful for such experiments, involve head-mounted systems that may also interfere or distract participants from having a natural interaction. Although this kind of experiments are appealing, accuracy and data extraction constraints require a transitory period until to find the more natural environment aiming to minimize interferences with the participant. Third, the implementation of paradigms where individuals interact with someone else in a meaningful manner could give useful information to elucidate processes related to the observed “someone else,” alone in the paradigm, or in an interacting situation (Schilbach et al., 2013).

Finally, the present study allowed us to identify the influence of empathy trait and gender on empathic responses and associated recording eye movements during the inspection of emotional dynamic stimuli. In this sense, our results support the idea that a high empathy trait has an effect on the affective and cognitive empathic responses, regardless of gender, but it does not affect the emotional recognition component. Likewise, we found a negative relation between empathy trait and symptoms of alexithymia, which could affect the empathic responses. Otherwise, the fact that women spent more time on the eyes area did not seem to affect the empathic responses to the dynamic emotional stimulus. Overall, empathic responses of both men and women are modulated by their empathic trait. In addition, empathic trait and gender seem to impact strategies to deal with emotional facial information. The originality of present research opens new avenues to try to disentangle links between empathy components and facial expressions of emotion, natural windows for social interactions.

## Data Availability Statement

The datasets generated for this study are available on request to the corresponding author.

## Ethics Statement

The studies involving human participants were reviewed and approved by Facultad de Psicología BUAP-098082. The patients/participants provided their written informed consent to participate in this study.

## Author Contributions

EM-V contributed to the study on conception and design, the analysis and interpretation of data, the manuscript writing and supervised. AG contributed to the manuscript writing, the acquisition, and the analysis of data. YC reviewed the manuscript and provided critical revisions. HS contributed to the study on interpretation of data and supervised the manuscript writing. All authors approved the final version of the manuscript for submission.

### Conflict of Interest

The authors declare that the research was conducted in the absence of any commercial or financial relationships that could be construed as a potential conflict of interest.
